# Site Directed Mutagenesis of *Schizosaccharomyces pombe* Glutathione Synthetase Produces an Enzyme with Homoglutathione Synthetase Activity

**DOI:** 10.1371/journal.pone.0046580

**Published:** 2012-10-16

**Authors:** Tamara Dworeck, Martin Zimmermann

**Affiliations:** 1 Department of Biology, RWTH Aachen University, Aachen, Germany; 2 Institute of Biologie IV- Applied Microbiology, RWTH Aachen University, Aachen, Germany; Institute of Molecular Genetics IMG-CNR, Italy

## Abstract

Three different His-tagged, mutant forms of the fission yeast glutathione synthetase (GSH2) were derived by site-directed mutagenesis. The mutant and wild-type enzymes were expressed in *E. coli* DH5α and affinity purified in a two-step procedure. Analysis of enzyme activity showed that it was possible to shift the substrate specificity of GSH2 from Gly (k_m_ 0,19; wild-type) to β-Ala or Ser. One mutation (substitution of Ile471, Cy472 to Met and Val and Ala 485 and Thr486 to Leu and Pro) increased the affinity of GSH2 for β-Ala (k_m_ 0,07) and lowered the affinity for Gly (k_m_ 0,83), which is a characteristic of the enzyme homoglutathione synthetase found in plants. Substitution of Ala485 and Thr486 to Leu and Pro only, increased instead the affinity of GSH2 for Ser (k_m_ 0,23) as a substrate, while affinity to Gly was preserved (k_m_ 0,12). This provides a new biosynthetic pathway for hydroxymethyl glutathione, which is known to be synthesized from glutathione and Ser in a reaction catalysed by carboxypeptidase Y. The reported findings provide further insight into how specific amino acids positioned in the GSH2 active site facilitate the recognition of different amino acid substrates, furthermore they support the evolutionary theory that homoglutathione synthetase evolved from glutathione synthetase by a single gene duplication event.

## Introduction

Glutathione (γGluCys-Gly; GSH) is a low-molecular-weight thiol found in most eukaryotic and prokaryotic organisms. It is thought to protect intracellular structures such as proteins, membranes and nucleic acids against oxidative damage caused by *e.g.* hydrogen peroxide. Additionally it maintains a reducing thiol/disulfide-balance in the cell and facilitates the detoxification of foreign compounds [1]–[3].

There are at least three GSH homologues found in the plant kingdom, which perform many of the functions described above for GSH. The tripeptide homo-glutathione (γGluCys-β-Ala; hGSH) is contained in Fabaceae [4], whereas the Poaceae family contains hydroxymethyl-glutathione (γGluCys-Ser; hmGSH) [5]. Another GSH homologue harbours a terminal Glu and was first isolated from maize seedlings exposed to cadmium [6]. The occurrence of the different GSH homologues in plants seems to be mainly due to the acyl acceptor availability, in maize and wheat for instance Gly is accessible, while soya beans contain more β-Ala [7].

Most of these tripeptides are thought to be synthesized enzymatically from their constituent amino acids. In most cases this begins with the formation of the dipeptide γGluCys, catalysed by γGluCys synthetase. GSH synthetase catalyzes the second step in the formation of GSH, which is the ATP-dependant condensation of γGluCys and Gly [8]. The hmGSH is known to be synthesized enzymatically from GSH in a reaction catalysed by carboxypeptidase Y [9] although other synthetic pathways may exist, as Skipsey *et al.* (2005) suggest that plant GSH synthetases have a flexible amino acid substrate specificity that enables them to synthetase hmGSH directly from γGluCys and Ser [7]. In contrast, hGSH is known to be synthesized by hGSH synthetase, which has been isolated from leguminous plants such as *Medicago trunculata* and *Vigna radiata* [10], [11].

The hGSH-synthetase is very similar to GSH-synthetase in function and primary sequence, but both enzymes differ in their substrate specificity. GSH-synthetase binds with high specificity to Gly, whereas hGSH-synthetase shows a higher affinity towards β-Ala, but does also accept Gly as a substrate. Due to the abovementioned sequence similarities, it has been suggested that the corresponding gene *gsh2* (hGSH) arose from a gene duplication event involving the *gsh1* ORF (GSH). This evolutionary event occurred after the divergence of the Fabales, Solanales and Brassicales. Consistent with this model it has been possible to construct a more GSH-synthetase-like enzyme by substituting both Leu534 and Pro535 with Ala in the *M. trunculata* hGSH-synthetase [10]. The corresponding amino acid residue of the human GSH synthetase is thought to be positioned within the glycyl-binding site [12].

Fission yeast GSH synthetase (GSH2), whose sequence and function are well-characterized [13], is like all other GSH synthetases highly specific for Gly. Therefore, it is interesting to ask whether it is possible to change the enzyme specificity from the original amino acid substrate to one or even several different amino acids by mutagenesis.

Here we report the heterologous expression of several different His-tagged, mutated forms of the fission yeast GSH synthetase, constructed by site-directed mutagenesis. The target positions to be mutated (Ile471, Cys472, Ala485 and Thr486) were chosen after comparing the sequences of the known hGSH-synthetases and GSH-synthetases. The mutated genes were expressed in *Escherichia coli*. The enzymes were affinity purified and their *in vitro* activities and kinetic properties were determined. It has been possible to construct mutant enzymes with changed substrate affinity, *e.g.* an enzyme with more hGSH-like characteristics as well as one which acccepts Ser as a substrate. Furthermore the experiments provided new data concerning the function of different amino acid residues in the enzyme primary sequence regarding substrate binding and differential recognition of Gly, β-Ala and Ser. Moreover presented data support the theory of a gene duplication event leading to the development of hGSH synthetase starting out from a GSH synthetase during evolution.

## Materials and Methods

### DNA methods

Standard molecular biology techniques were used for DNA isolation, analysis and cloning [14]. The identity of all clones was verified by DNA sequencing, carried out by SequiServe (Vaterstetten, Germany) following the method of Sanger.

### Site-directed mutagenesis

**Figure 1:**
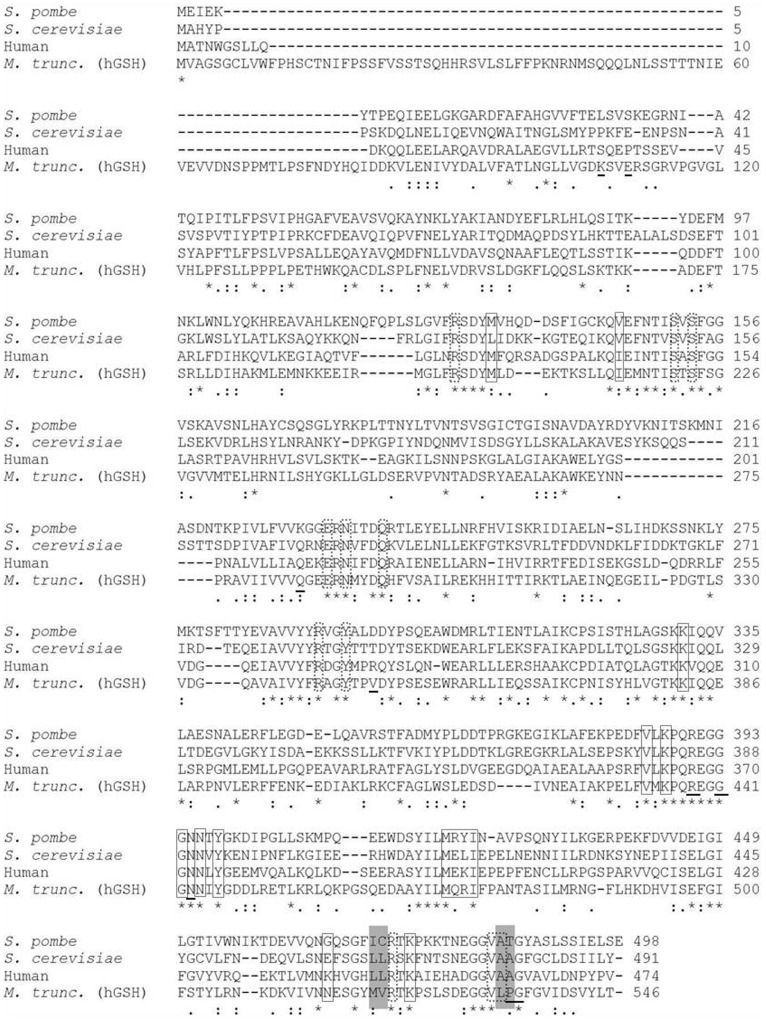
Multiple amino acid sequence alignment of the GSH synthetases from *S. pombe, S. cerevisiae* and *H. sapiens* with the hGSH synthetase sequence of *M. trunculata*. SWISSPROT-TREMBL accession numbers are as follows: P35669 (GSH synth. *S. pombe*), Q08220 (GSH synth. *S. cerevisiae*), P48637 (GSH synth. human) and AF194422 (hGSH synth. *M. trunculata*). Dots represent conserved amino acid residues, whereas stars represent identical residues. Ile471, Cys472, Ala485 and Thr486 (fission yeast GSH synthetase) and corresponding residues in the other enzymes are highlighted in grey. Squares – amino acid residues that are part of the ATP binding-site; dotted squares – amino acid residues that are part of the GSH binding site; underlined – amino acids within the entrance of the binding pocket.

Site-directed mutagenesis was carried out using the GeneTailor™ Site-Directed Mutagenesis System kit provided by Invitrogen (Carlsbad, USA) using pTrc99A-GSH2 or pTrc99A-GSH2-at/lp as template. Primers were purchased from Metabion (Martinsried, Germany). Table 1 shows all oligonucleotides used, while Table 2 shows the corresponding templates and resulting GSH2-mutants (B). Three different mutated *gsh2* ORFs were constructed. After transforming *E. coli* DH5α with the different, amplified *gsh2*-pTrc99A constructs and selective screening for positive clones, the isolated plasmid DNAs were sequenced to verify whether the correct base substitutions and no other unwanted mutations had been introduced. Two double mutant enzymes were created. The first carried the amino acids Met and Val instead of Ile471 and Cys472, whereas in the second one Ala485 and Thr486 were substituted with Leu and Pro. In the final GSH2 mutant, all of the above mentioned amino acid substitutions were combined. The positions were chosen after analysing the amino acid sequence alignment shown in Fig. 1, as they differ between the analyzed GSH synthetases and hGSH synthetase and might be involved in the binding of the amino acid substrates of GSH and hGSH synthetase (i.e. Gly and β-Ala) [11]. The mutant enzymes were expressed under control of the IPTG-inducible *trc*-promoter using the expression vector pTrc99A.

**Table 1:** Oligonucleotides and vector templates used for site-directed mutagenesis.

A. Oligonucleotide	Sequence (5′–3′)
GSH-ATLP-fw	AA ACT AAT GAA GGT GGT GTT **CTA CCA** GGC TAT GCT TCT
GSH2-ATLP-rev	AAC ACC ACC TTC ATT AGC TTT TTT GGG TTT
GSH2-ICMV-fw	AAA ATG GAC AGT CGG GTT TC**A TGG TA**C GTA CCA AAC C
GSH2-ICMV-rev	GAA ACC CGA CTG TCC ATT TTG AAC GAC TTC

Oligonucleotides and their sequences. [bold: mutated nucleotide positions].

**Table 2:** Oligonucleotides and vector templates used for site-directed mutagenesis.

B. Oligo. pair	Template	Resulting mutant
GSH-ICMV-fw/-rev	pTrc99A-GSH2	GSH2-IC/MV: Ile471, Cys472 substituted by Met471, Val472
GSH-ATLP-fw**/**-rev	pTrc99A-GSH2	GSH2-AT/LP: Ala485, Thr486 substituted by Leu485, Pro486
GSH-ICMV-fw**/**-rev	pTrc99A-GSH2-ATL	GSH2-AT/LP-IC/MV: all four substitutions present

Oligonucleotide pairs used for site-directed mutagenesis, as well as the corresponding templates and resulting amino acid substitutions. The source template pTrc99A-GSH2 consists of the *gsh2* ORF (including an 18-bp sequence encoding six His residues fused to the 3′ end of the gene) included in the *E. coli* expression vector pTrc99A.

### Bacterial strains and cultivation

*E. coli* DH5α was used for all DNA cloning experiments. Bacteria were cultivated in LB medium or LB medium containing 125 µg/ml of the antibiotic ampicillin. Transformation and plasmid isolation were carried out according to standard protocols [14].

### Protein Expression and Purification

*E. coli* cells were grown in 2×500 ml LBA at 28°C with constant agitation. The low temperatures resulted in slower cell growth and thus better expression of the heterologous proteins. For induction, cultures were supplemented with 1 mM IPTG after 3 h incubation. Cells were then harvested by centrifugation (10 min, 8000 g, GSA rotor, 4°C), washed twice with distilled water, resuspended in 10 ml of immobilized metal ion affinity chromatography (IMAC) binding buffer (20 mM Na_2_HPO_4_/Na_2_H_2_PO_4_, 500 mM NaCl, 10 mM imidazole, pH 7.4) and disrupted by treatment with lysozyme (10 mg/ml). After centrifugation (20 min, 12000 g, 4°C) the supernatant was purified as follows, with all steps carried out at 4°C. For IMAC, chelating Sepharose fast flow was loaded with nickel as described by the manufacturer (Amersham Pharmacia Biotech, Uppsala, Sweden). After applying the cell extract to the column and washing with binding buffer, bound protein was eluted by gradually increasing the imidazole concentration from 10 mM to 500 mM. GSH-synthetase eluted at an imidazole concentration of 200 mM. For further purification by reactive dye affinity chromatography, the protein sample was adjusted to the appropriate buffer (10 mM Tris-HCl, 20 mM NaCl, pH 7.8) by Sephadex G-25 gel (Amersham Pharmacia Biotech, Uppsala, Sweden) filtration chromatography and applied to a column packed with Cibacron blue 3GA-agarose (Sigma-Aldrich, Steinheim, Germany). After washing with 10 ml of buffer (10 mM Tris-HCl, 20 mM NaCl, pH 7.8), the elution was carried out by increasing the NaCl concentration in the same buffer to 250 mM. The concentration of purified protein was determined using the method of Lowry [15]. Purified proteins were verified by SDS-PAGE following the Laemmli protocol [16] using the VIIL molecular mass standard (Sigma-Aldrich, Steinheim, Germany).

### GSH-synthetase activity assay

Purified GSH-synthetase and its mutant forms were equilibrated to 100 mM Tris-HCl buffer, pH 8.2, by Sephadex G-25 gel filtration chromatography. GSH-synthetase activity was assayed according to Huang *et al.* [17] using γGluCys trifluoroacetate salt (Sigma-Aldrich, Steinheim, Germany) as a substrate (see also Fig. S1). One unit of enzyme is defined as the amount that catalyzes the formation of 1 µM of product per min. Protein concentrations were determined according to Lowry [15]. For V_max_ and k_m_ determination, the concentrations of two substrates were kept at saturating levels, while the third was varied. The concentrations of ATP and γGluCys were 0.002, 0.005, 0.01, 0.1, 0.5, 1.0 and 1.5 mM, and the concentrations of Gly (respectively β-Ala or Ser) were 0.1, 0.5, 1.0, 2.5, 5.0, 7.5 and 10.0 mM. Data was plotted according to Eadie-Hofstee, following the linearized Michaelis-Menten equation:
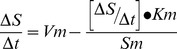



### Molecular modelling of the GSH2 mutant enzymes

Homology modelling of the three GSH2 mutants and the wild-type enzyme were carried out with the help of the I-TASSER server [18] which is available at the following URL: http://zhanglab.ccmb.med.umich.edu/I-TASSER. The models were then modified and visualized using VMD (Visual Molecular Dynamics program ver. 1.6, http://www.ks.uiuc.edu/Research/vmd/).

## Results

### Comparison of the primary sequences of GSH synthetase and hGSH synthetase

Amino acid sequences of three different GSH synthetases (from human, fission yeast and *Saccharomyces cerevisiae*) were compared with *M*. *trunculata* hGSH synthetase in a multiple sequence alignment using the EMBL-EBI CLUSTAL W Server [19]. The purpose was to identify amino acids positioned in the active site of each enzyme, as these would be the most likely to play a role in the differential recognition of amino acid substrates and would probably differ between GSH and hGSH. Fig. 1 shows the alignment result. All three GSH synthetases show strongly conserved sequences and a high level of sequence identity with hGSH synthetase. Major differences between the analysed GSH synthetases and the hGSH synthetase were found in positions 471 and 472, which are positioned within the substrate binding pocket. A further difference was found at the positions 485 and 486 that sit at the entrance of the binding pocket and are involved in amino acid substrate recognition in the human GSH2 [12]. Fendo *et al*. reported that a within the hGSH of *M. trunculata* a change of the amino acids at that position (Leu and Pro) to a double Ala led to a partial change of substrate recognition from ß-Ala to Gly [11]. All four positions were therefore chosen for site-directed mutagenesis.

### Cloning, expression and purification of the different GSH2-mutants

Two double mutant GSH2 and one quadruple mutant enzyme were created. The first one carrying the amino acids Met and Val instead of Ile471 and Cys472 (GSH2-IC/MV), whereas in the second one Ala495 and Thr486 (GSH2-AT/LP) were substituted by Leu and Pro. In the final GSH2 mutant, all of the above mentioned amino acid substitutions were combined (GSH2-IC/MV-AT/LP). All His-tagged GSH2 mutants were expressed in *E. coli* DH5α and purified to apparent homogeneity in a two-step affinity chromatography procedure. During the first purification step (IMAC) the target proteins co-eluted with a few other proteins. These contaminations were removed during the subsequent reactive dye affinity chromatography. Protein purity was 95% as determined by ImageJ analysis [20].

**Figure 2:**
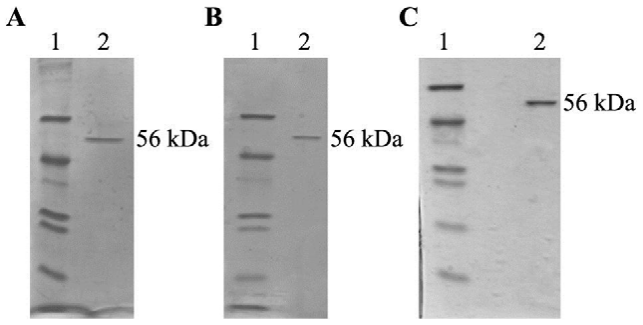
SDS-PAGE analysis of the different mutated forms of the fission yeast GSH synthetase after purification. Part A shows GSH2-IC/MV, Part B shows GSH-AT/LP and Part C shows GSH2AT/LP-IC/MV. In all cases, the first lane shows the molecular mass standard VIIL with bands of 66, 45, 36, 29, 24, 20 and 14.2 kDa, while the second lane shows the purified GSH2 enzymes (56 kDa).

SDS-PAGE analysis revealed a single band of approximately 56 kDa for each purified GSH2 mutant (Fig. 2). As a comparison, the wild-type enzyme was expressed and purified in the same way (data not shown).

### Comparison of the mutant and wild-type enzymes

The specific activities of the purified His-tagged proteins were determined using three different amino acids as potential substrates (Gly, β-Ala, Ser). Furthermore, the K_m_-values and specificity factors for the amino acid(s) exchanged by each enzyme were calculated after obtaining Eadie-Hofstee plots of the kinetic data (all plots are reported in the Fig. S2, S3, S4, S5, S6, S7). All results are summarized in Table 3. The wild-type fission yeast GSH synthetase used Gly as a substrate, but not β-Ala or Ser. In comparison, the GSH2-IC/MV mutant showed a much lower affinity towards Gly and a lower activity (∼22% of wild-type activity) but no other differences. Mutation of the second position, substitution of Ala485 and Thr486 by Leu and Pro, resulted in lower activity with Gly (∼30% of wild-type activity) and a shift towards the acceptance of Ser. However, the enzyme was 8.6-fold more specific for Gly as compared to Ser. GSH2-IC/MV-AT/LP, in which all four relevant amino acids were substituted, showed a slightly decreased activity with Gly (62% of the wild-type activity) and was able to use β-Ala as a substrate, thus gaining the main characteristic of hGSH synthetase. The enzyme was more specific for β-Ala than for Gly (1.2-fold higher specificity). Additionally the amino acid Glu had been tested as a possible substrate for the GSH2 variants, as in plants there are known phytochelatins containing Glu instead of Gly, however no activity was detected (results not shown).

**Table 3:** Enzyme activities and substrate specificities of GSH2 mutants and wild-type GSH synthetase.

Protein	AS substrate	V_max_ (U/mg)	K_m_-values	Specificity factors (V_max_/K_m_)
GSH2 (wild-type)	β-Ala	0	-	-
	Gly	2.74 (100%)	0.19	14.42
	Ser	0	-	-
GSH2 (IC/MV)	β-Ala	0	-	-
	Gly	0.60 (21. 9%)	0.46	1.30
	Ser	0	-	-
GSH2 (AT/LP)	β-Ala	0	-	-
	Gly	0.82 (29.9%)	0.12	6.83
	Ser	0.18	0.23	0.78
GSH2 (IC/MV-AT/LP)	β-Ala	0.17	0.07	2.43
	Gly	1.70 (62.0%)	0.83	2.05
	Ser	0	-	-

All enzymes showed Michaelis-Menten kinetics with the tolerated amino acid substrates. The V_max_ value for wild-type GSH synthetase was defined arbitrarily as 100% such that the V_max_ values of the mutant enzymes (with Gly as a substrate) were expressed as a percentage of the wild-type value.

In contrast to the wild-type enzyme the mutated variants were difficult to isolate and performed poorly when produced in the *S. pombe*. This might be due to *in vivo* protein cleavage as it is known that GSH2 *in vitro* is cleaved into two parts by a metallo-protease between Ala217 and Ser218 [21] and is active as a hetero tetramer as well as in the non-cleaved homodimeric form. Results of another study revealed that this cleavage might have an influence on the differential substrate recognition in the way that the cleaved enzyme accepts Gly only (unpublished data not shown).

### Homology modelling of GSH2 wild-type and variants

**Figure 3:**
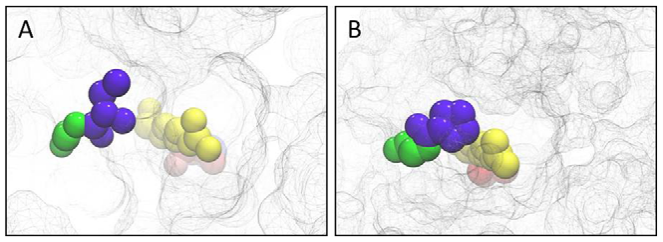
Wireframe surface representation of microenvironment around the amino acid positions (VdW representation) 471, 472, 485 and 486 in the GSH2 wild-type (A) and GSH2-IC/MV-AT/LP (B). A – GSH2 wild-type: Ile471 (red), Cys472 (yellow), Ala485 (blue) and Thr486 (green); B – GSH2-IC/MV-AT/LP: Met471 (red), Val472 (yello), Leu485 (blue), Pro486 (green).

Structural models of GSH2-IC/MV-AT/LP and wild-type GSH2 were obtained based on known crystal structures (*e.g.* human, *S. cerevisiae*, *T. brucei* GSH synthetase). The estimated accuracy of the model was 0.93±0.06 (TM-score). To get an idea of the location of the mutated amino acid positions wireframe surface representations of the surrounding of the GSH2 binding pocket with VdW representation of considered positions are given in Fig. 3. It can be seen that positions 471 and 472 are within the substrate binding pocket, while 485 and 486 sit at the channel entrance.

Besides the models show that wild-type and mutant enzymes differ mainly in the number of hydrogen bonds the four relevant amino acid residues backbones are able to form with the backbones of the surrounding amino acids (see Fig. S8). Ile (471, GSH2 wild-type) forms two hydrogen bonds with Gly448, whereas Cys (472, GSH2 wild-type) forms two hydrogen bonds with Ser492. In comparison, the Met residue found at position 471 in the mutant GSH2-IC/MV and GSH2-IC/MV-AT/LP forms two hydrogen bonds with Gly448, whereas Val472 interacts with Ser492 via only one hydrogen bond. Ala (485, GSH2 wild-type) does not form any hydrogen bonds with surrounding amino acids, whereas Thr (486, GSH2 wild-type) shows one hydrogen bond with Gly483 and two with Lys478. In the mutant enzyme GSH-AT/LP and GSH2-IC/MV-AT/LP Ala485 and Thr486 are substituted by Leu and Pro. In this case only Pro is able to form hydrogen bonds, *i.e.* one bond with Lys478.

Local secondary structure changes only in case of a substitution of Thr486 by Pro (from β-sheet to random coil), while the other mutations keep the secondary structure found for the wild-type amino acids, as seen in a Ramachandran plot of the four analysed positions (see Fig. S9).

## Discussion

### GSH and hGSH substrate specificity

GSH synthetase is found in most eukaryotes, and is highly specific for Gly. In contrast, the hGSH synthetase found in plants is much more specific for β-Ala, although it also accepts Gly as a substrate. It has been possible to create an enzyme with similar characteristics to GSH synthetase from *M. trunculata* hGSH synthetase by substituting both Leu534 and Pro535 with Ala [10], which are conserved in GSH synthetase and can be found for instance in *S. cerevisiae* and human GSH synthetase at the corresponding amino acid positions. Thus both positions are thought to be important residues that play a role in the differential recognition the amino acid substrate. This was consistent with the findings of Polekhina *et al.* [12], who proposed that Ala462 is included in the glycyl-binding site of human GSH synthetase.

Therefore it is an interesting question whether it is possible to carry out the opposite process, *i.e.* to change the well-characterized fission yeast GSH synthetase into an enzyme with the substrate specificity of an hGSH. As shown in the sequence alignment results (Fig. 1) positions 471, 472, 485 and 486 were found to be interesting candidates for mutagenesis as they are positioned within the substrate binding pocket (471, 472) or near to the pocket entrance (485, 486) and are known to play a role in substrate recognition. The aforementioned positioned were to be changed to the amino acids found in *M. trunculata* hGSH synthetase. Both double mutants GSH2-IC/MV, GSH2- AT/LP, as well as the quadruple mutant GSH2-IC/MV-AT/LP were constructed. The results show that only a mutation at all four positions lead to the desired effect, broadening substrate recognition from Gly to β -Ala, with an overall higher specificity for β-Ala.

We therefore conclude that also in the fission yeast *S. pombe* the four amino acids are likely to play a role in the amino acid substrate recognition process. This presumption is supported by the fact that mutant GSH2-AT/LP tolerated Ser besides the usual substrate Gly. In addition this result suggests the possible existence of a yet-to-be discovered enzyme, which is able to catalyze the formation of hmGSH from γ-GlyCys and Ser directly instead of exchanging Gly in GSH to Ser, as catalysed by carboxypeptidase Y [8].

To get first insights into the changes at the molecular level, homology models of all GSH2 variants, as well as the wild-type were built (see Fig. 3 and Supp. Info. Fig. S1). Modelling shows that the introduced amino acid substitutions predominantly affect the number of backbone to backbone hydrogen bonds and have an effect on the secondary structure only in case of the substitution of Thr486 to Pro (see Fig. S2). Compared to Ile471, Cys472, Ala485 and Thr486 in wild-type GSH2, the amino acids found at the same positions in the three GSH2 mutants interact with fewer surrounding amino acid residues by hydrogen bond formation. Possibly this loss of interaction results in a less constrained enzyme conformation and might lead to a higher structural flexibility.

Overall the presented results support the phylogenetic findings of Frendo *et al.* [11], which suggest that hGSH synthetase evolved by way of a tandem gene duplication that took place after the divergence of Fabales, Solanales and Brassicales, as it has been possible to construct an hGSH synthetase like enzyme by specifically mutating fission yeast GSH2. Furthermore, our mutant GSH2-AT/LP, which uses Ser as a substrate, shows that a hypothetical hmGSH synthetase could have evolved of a GSH synthetase in a similar way.

## Supporting Information

Figure S1 **Reaction scheme of the assay used to monitor GSH-sythetase activity.** GSH-synthetase activity was assayed according to Huang, C., He, W., Meister, A. and Anderson, M. (1995) *Proc Natl Acad Sci*. USA **92**, 1232–1236.
(JPG)

Figure S2 **Eadie-Hofstee Plot of kinetic data obtained for GSH2 wild type.** The concentrations of the amino acid substrate (Gly) were 0.1, 0.5, 1.0, 2.5, 5.0, 7.5 and 10.0 mM.
(JPG)

Figure S3 **Eadie-Hofstee Plot of kinetic data obtained for GSH2 mutant AT/LP.** The concentrations of the amino acid substrate (Gly) were 0.1, 0.5, 1.0, 2.5, 5.0, 7.5 and 10.0 mM.
(JPG)

Figure S4 **Eadie-Hofstee Plot of kinetic data obtained for GSH2 mutant AT/LP.** The concentrations of the amino acid substrate (Ser) were 0.1, 0.5, 1.0, 2.5, 5.0, 7.5 and 10.0 mM.
(JPG)

Figure S5 **Eadie-Hofstee Plot of kinetic data obtained for GSH2 mutant IC/MV.** The concentrations of the amino acid substrate (Gly) were 0.1, 0.5, 1.0, 2.5, 5.0, 7.5 and 10.0 mM.
(JPG)

Figure S6 **Eadie-Hofstee Plot of kinetic data obtained for GSH2 mutant IC/MV-AT/LP.** The concentrations of the amino acid substrate (Gly) were 0.1, 0.5, 1.0, 2.5, 5.0, 7.5 and 10.0 mM.
(JPG)

Figure S7 **Eadie-Hofstee Plot of kinetic data obtained for GSH2 mutant IC/MV-AT/LP.** The concentrations of the amino acid substrate (β-Ala) were 0.1, 0.5, 1.0, 2.5, 5.0, 7.5 and 10.0 mM.
(JPG)

Figure S8 **Ball and stick models of the microenvironment around the amino acid positions 471, 472, 485 and 486 in the GSH2 mutants and the wild type enzyme.** A – GSH2-IC/MV: Met471 (red), Val472 (green); B – GSH2-AT/LP: Leu485 (yellow), Pro486 (purple)**;** C – GSH2-IC/MV-AT/LP: Met471 (red), Val472 (green), Leu485 (yellow), Pro486 (purple); D – GSH2-WT (position 1): Ile471 (red), Cys472 (green); E – GSH2-WT (position 2): Ade485 (yellow), Thr486 (purple). Residues that form hydrogen bonds (black lines) with the relevant amino acids are labelled in black, and corresponding bond lengths (in Å) are labelled in red. O atoms are displayed as red, N atoms as blue and C atoms as grey. Structural modelling of the three GSH2 mutants and the wild type enzyme were carried out with the help of the Swiss-Model server (Schwede, T., Kopp, J., Guex, N. and Peitsch, C. (2003) *Nucleic Acids Res*. **31**, 3381–3385) which is available over the Internet at the following URL: http://swissmodel.expasy.org. The models were then modified and visualized using RasMol 2.7 (3.Sayle, R. A. and Milner-White, E. J. (1996) Trends Biochem. Sci. 20, 374–376).
(JPG)

Figure S9 **Local Ramachandran Plots of positions 471, 472, 485 and 486 in the GSH2 quadruple mutant and the wild type enzyme.** A – GSH2-WT B – GSH2-IC/MV-AT/LP Local Ramachandran plots for Ile471, Cys472, Ala485 and Thr486 in the wild type GSH2 and Met471, Val472, Leu485 and Pro486 in GSH2-IC/MV-AT/LP have been plotted using VMD (Visual Molecular Dynamics program ver. 1.6, http://www.ks.uiuc.edu/Research/vmd/), based on the models mentioned in figure S8.
(JPG)
