# Supplementary figures and images for: Site Directed Mutagenesis of Schizosaccharomyces pombe Glutathione Synthetase Produces an Enzyme with Homoglutathione Synthetase Activity
Source: PLoS One. 2012 Oct 16;7(10):e46580. doi: 10.1371/journal.pone.0046580 (PMC3473041; doi:10.1371/journal.pone.0046580)

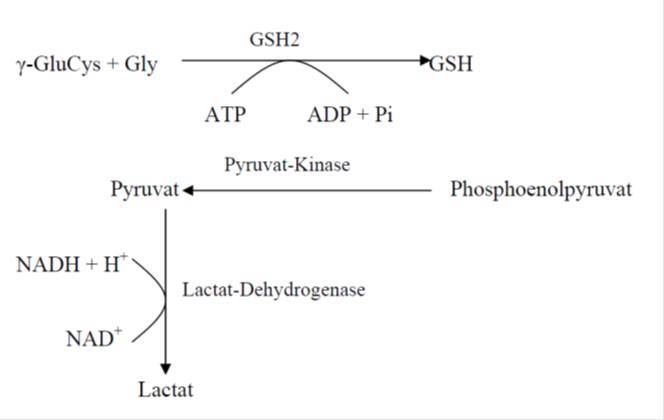

Supplement: Figure S1 — Reaction scheme of the assay used to monitor GSH-sythetase activity. GSH-synthetase activity was assayed according to Huang, C., He, W., Meister, A. and Anderson, M. (1995) Proc Natl Acad Sci. USA 92, 1232–1236. (JPG) [file pone.0046580.s001.jpg]

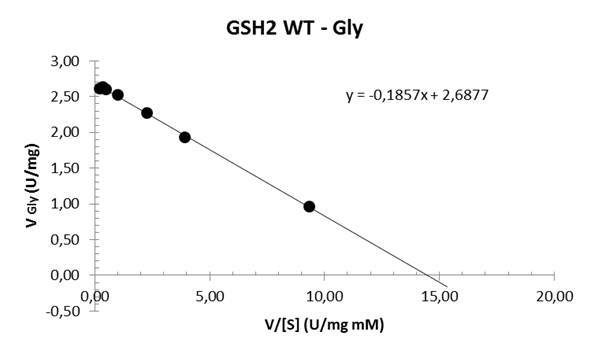

Supplement: Figure S2 — Eadie-Hofstee Plot of kinetic data obtained for GSH2 wild type. The concentrations of the amino acid substrate (Gly) were 0.1, 0.5, 1.0, 2.5, 5.0, 7.5 and 10.0 mM. (JPG) [file pone.0046580.s002.jpg]

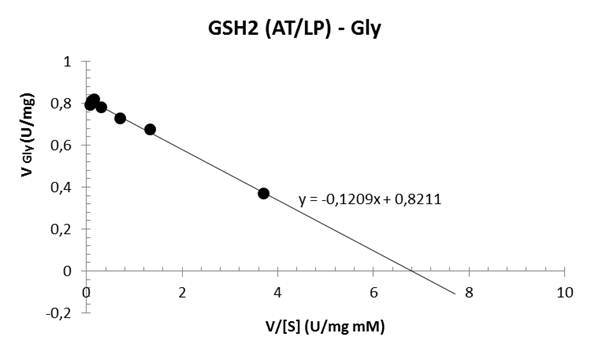

Supplement: Figure S3 — Eadie-Hofstee Plot of kinetic data obtained for GSH2 mutant AT/LP. The concentrations of the amino acid substrate (Gly) were 0.1, 0.5, 1.0, 2.5, 5.0, 7.5 and 10.0 mM. (JPG) [file pone.0046580.s003.jpg]

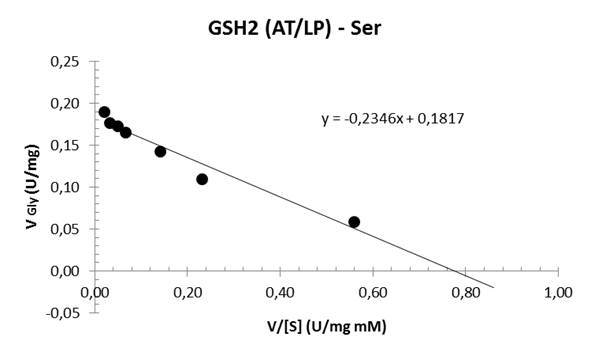

Supplement: Figure S4 — Eadie-Hofstee Plot of kinetic data obtained for GSH2 mutant AT/LP. The concentrations of the amino acid substrate (Ser) were 0.1, 0.5, 1.0, 2.5, 5.0, 7.5 and 10.0 mM. (JPG) [file pone.0046580.s004.jpg]

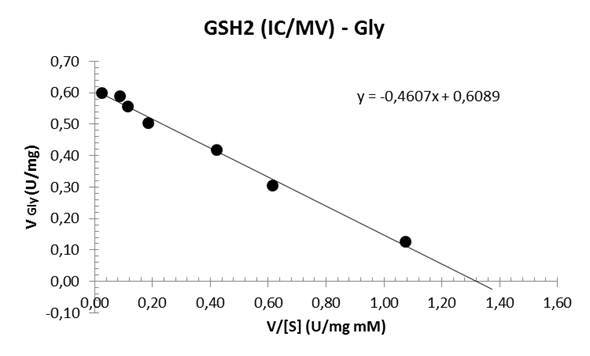

Supplement: Figure S5 — Eadie-Hofstee Plot of kinetic data obtained for GSH2 mutant IC/MV. The concentrations of the amino acid substrate (Gly) were 0.1, 0.5, 1.0, 2.5, 5.0, 7.5 and 10.0 mM. (JPG) [file pone.0046580.s005.jpg]

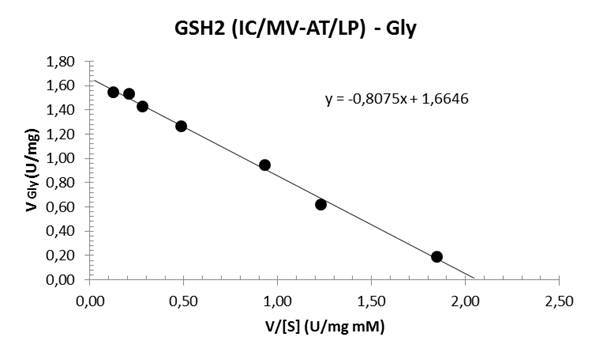

Supplement: Figure S6 — Eadie-Hofstee Plot of kinetic data obtained for GSH2 mutant IC/MV-AT/LP. The concentrations of the amino acid substrate (Gly) were 0.1, 0.5, 1.0, 2.5, 5.0, 7.5 and 10.0 mM. (JPG) [file pone.0046580.s006.jpg]

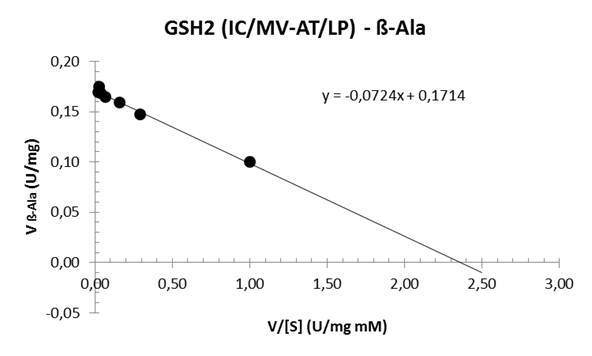

Supplement: Figure S7 — Eadie-Hofstee Plot of kinetic data obtained for GSH2 mutant IC/MV-AT/LP. The concentrations of the amino acid substrate (β-Ala) were 0.1, 0.5, 1.0, 2.5, 5.0, 7.5 and 10.0 mM. (JPG) [file pone.0046580.s007.jpg]

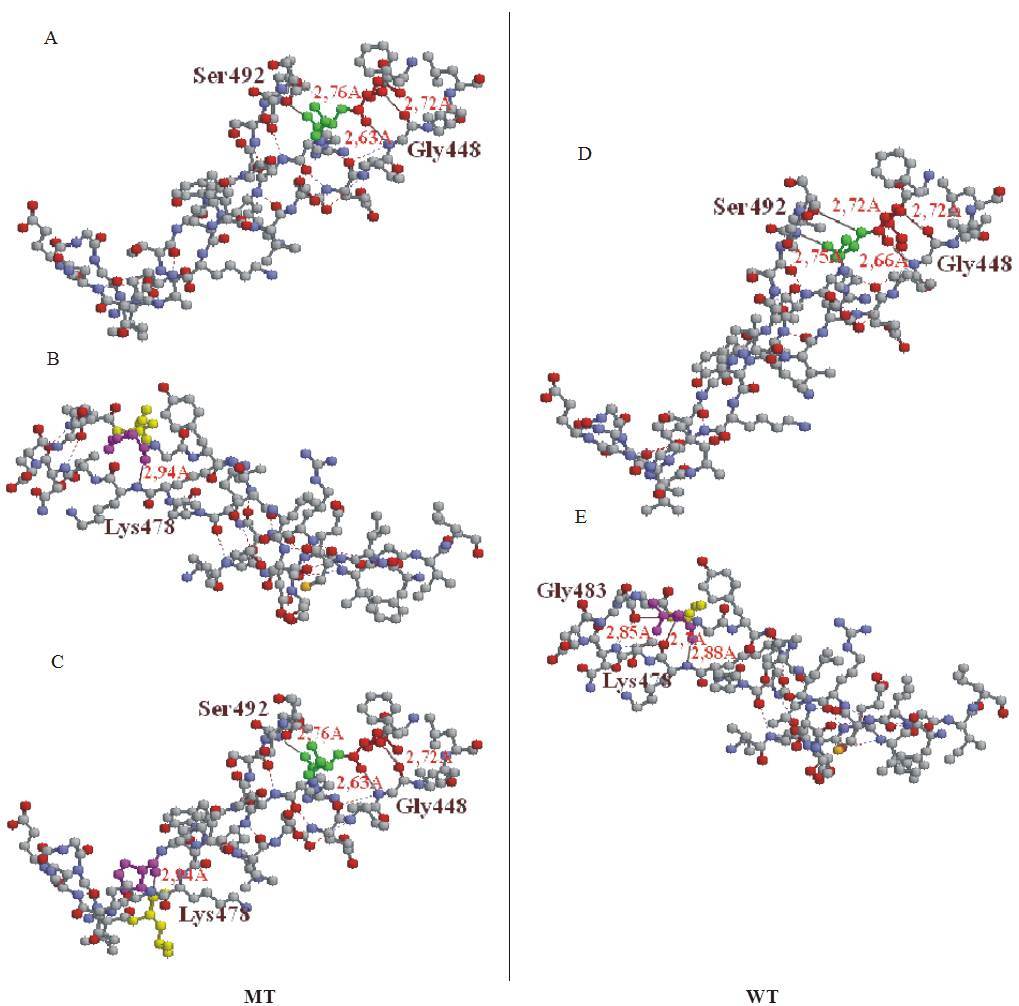

Supplement: Figure S8 — Ball and stick models of the microenvironment around the amino acid positions 471, 472, 485 and 486 in the GSH2 mutants and the wild type enzyme. A – GSH2-IC/MV: Met471 (red), Val472 (green); B – GSH2-AT/LP: Leu485 (yellow), Pro486 (purple); C – GSH2-IC/MV-AT/LP: Met471 (red), Val472 (green), Leu485 (yellow), Pro486 (purple); D – GSH2-WT (position 1): Ile471 (red), Cys472 (green); E – GSH2-WT (position 2): Ade485 (yellow), Thr486 (purple). Residues that form hydrogen bonds (black lines) with the relevant amino acids are labelled in black, and corresponding bond lengths (in Å) are labelled in red. O atoms are displayed as red, N atoms as blue and C atoms as grey. Structural modelling of the three GSH2 mutants and the wild type enzyme were carried out with the help of the Swiss-Model server (Schwede, T., Kopp, J., Guex, N. and Peitsch, C. (2003) Nucleic Acids Res. 31, 3381–3385) which is available over the Internet at the following URL: http://swissmodel.expasy.org. The models were then modified and visualized using RasMol 2.7 (3.Sayle, R. A. and Milner-White, E. J. (1996) Trends Biochem. Sci. 20, 374–376). (JPG) [file pone.0046580.s008.jpg]

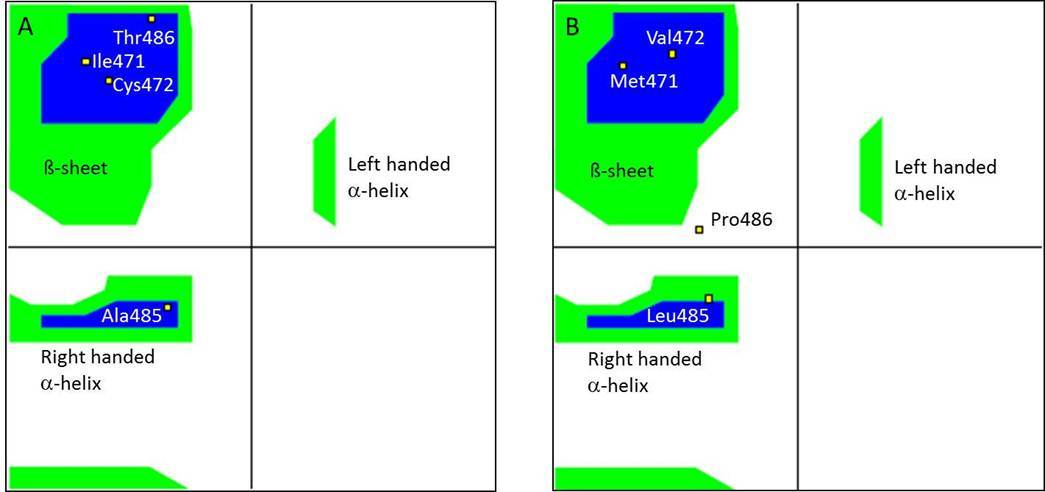

Supplement: Figure S9 — Local Ramachandran Plots of positions 471, 472, 485 and 486 in the GSH2 quadruple mutant and the wild type enzyme. A – GSH2-WT B – GSH2-IC/MV-AT/LP Local Ramachandran plots for Ile471, Cys472, Ala485 and Thr486 in the wild type GSH2 and Met471, Val472, Leu485 and Pro486 in GSH2-IC/MV-AT/LP have been plotted using VMD (Visual Molecular Dynamics program ver. 1.6, http://www.ks.uiuc.edu/Research/vmd/), based on the models mentioned in figure S8. (JPG) [file pone.0046580.s009.jpg]
